# Modulation of tumor fatty acids, through overexpression or loss of thyroid hormone responsive protein spot 14 is associated with altered growth and metastasis

**DOI:** 10.1186/s13058-014-0481-z

**Published:** 2014-12-04

**Authors:** Elizabeth A Wellberg, Michael C Rudolph, Andrew S Lewis, Nuria Padilla-Just, Paul Jedlicka, Steven M Anderson

**Affiliations:** 1 Department of Pathology, University of Colorado School of Medicine, University of Colorado Anschutz Medical Campus, Aurora, 80045 CO USA; 2 Program in Cancer Biology, University of Colorado School of Medicine, University of Colorado Anschutz Medical Campus, 12801 East 17th Avenue, Aurora, 80045 CO USA; 3 Division of Endocrinology, Metabolism, and Diabetes, Department of Medicine, University of Colorado School of Medicine, University of Colorado Anschutz Medical Campus, Aurora, 80045 CO USA; 4 Division of Medical Oncology, Department of Medicine, University of Colorado School of Medicine, University of Colorado Anschutz Medical Campus, Aurora, 80045 CO USA

## Abstract

**Introduction:**

Spot14 (S14), encoded by the *THRSP* gene, regulates *de novo* fatty acid synthesis in the liver, adipose, and lactating mammary gland. We recently showed that S14 stimulated fatty acid synthase (FASN) activity *in vitro*, and increased the synthesis of fatty acids in mammary epithelial cells *in vivo*. Elevated *de novo* fatty acid synthesis is a distinguishing feature of many solid tumors compared with adjacent normal tissue. This characteristic is thought to be acquired during tumor progression, as rapidly proliferating cells have a heightened requirement for membrane phospholipids. Further, overexpression of FASN is sufficient to stimulate cell proliferation. While many studies have focused on the FASN enzyme in cancer biology, few studies have addressed the roles of proteins that modify FASN activity, such as S14.

**Methods:**

Tumor fatty acids were modulated using two mouse models, mouse mammary tumor virus (MMTV)-neu mice overexpressing S14 and MMTV-polyomavirus middle T antigen (PyMT) mice lacking S14, and associations between elevated or impaired fatty acid synthesis on tumor latency, growth, metastasis, and signaling pathways were investigated. We evaluated S14-dependent gene expression profiles in mouse tumors by microarray and used publicly available microarray datasets of human breast tumors.

**Results:**

S14 overexpression in the MMTV-Neu transgenic model is associated with elevated medium-chain fatty acids, increased proliferation and a shorter tumor latency, but reduced tumor metastasis compared to controls. Loss of S14 in the MMTV-PyMT model decreased FASN activity and the synthesis of medium-chain fatty acids but did not alter tumor latency. Impaired fatty acid synthesis was associated with reduced solid tumor cell proliferation, the formation of cystic lesions in some animals, and decreased phosphorylation of Src and protein kinase B (Akt). Analysis of gene expression in these mouse and human tumors revealed a relationship between S14 status and the expression of genes associated with luminal epithelial differentiation.

**Conclusions:**

This study demonstrates a potential role for S14 in regulating mammary tumor growth and fatty acid synthesis *in vivo*. Furthermore, these results suggest that modulating the amount of medium chain fatty acids, by changing the levels of S14, has the potential to impact malignant mammary tumor phenotypes.

**Electronic supplementary material:**

The online version of this article (doi:10.1186/s13058-014-0481-z) contains supplementary material, which is available to authorized users.

## Introduction

Spot14 (S14), the protein product of the thyroid hormone responsive protein spot14 (*Thrsp*) gene, is consistently associated with regulation of the *de novo* fatty acid synthesis pathway. S14 was originally discovered as a protein that was induced in the liver of fasted rats stimulated by lipogenic factors [1]. Mice lacking the S14 gene produce milk with 60% lower levels of *de novo* synthesized fatty acids during lactation, compared to wild-type mice, and are resistant to diet-induced obesity [2]-[4]. We previously characterized gene expression profiles of the mouse mammary gland throughout pregnancy and lactation to identify factors that regulate the sharp increase in fatty acid synthesis observed at parturition [5]. This study of the mammary epithelium revealed that S14 expression was associated with lactogenic differentiation, and its expression mirrored the pattern of fatty acid synthesis enzymes, acetyl CoA carboxylase (ACC), ATP-citrate lyase (ACLY) and fatty acid synthase (FASN) during the transition from pregnancy to lactation. In addition to its role in normal mammary function, S14 was shown to positively regulate *de novo* fatty acid synthesis and cell proliferation in human breast cancer cells [6],[7]. We have recently demonstrated that mammary epithelial cell overexpression of S14 in transgenic mice increases the *de novo* fatty acids present in milk [4]. Furthermore, we showed that addition of recombinant S14 to the FASN enzyme *in vitro* reduces the apparent Km, increases the apparent V_max_, and increases the synthesis of medium chain fatty acids (MCFA) relative to FASN alone [4]. These observations provide a mechanistic explanation for the described biological effects of S14, particularly in the mammary epithelium during lactation.

Many solid tumors display increased fatty acid synthesis compared to adjacent normal tissue [8]. This is due in part to increased *Fasn* expression and presumably, increased activity of the FASN enzyme [9]. While it appears that elevated levels of FASN are acquired during tumor progression, its exact role in tumorigenesis is not clear [10]. Increased tumor *de novo* fatty acid synthesis is predicted to provide several advantages: First, cancer cells are rapidly dividing and have a heightened requirement for membrane phospholipid precursors. Additionally, certain fatty acids can regulate intracellular signaling pathways either by activating signaling molecules or via post-translational modification of pro-tumorigenic kinases, such as Ras and Src [11]-[13]. Finally, elevated fatty acid synthesis may provide a survival advantage for cancer cells, allowing them to store lipids, which could serve as an energy reserve during the metastatic process. Although the requirement of FASN for breast cancer cell survival and proliferation has been established *in vitro* and in xenograft models, it is not known whether elevated fatty acid synthesis is sufficient to promote cancer metastasis [8],[10]. S14 represents a family of proteins that does not have enzymatic activity, but that modulates the activity of *de novo* fatty acid synthesis pathway enzymes to alter cellular physiology [4],[6],[14],[15].

In human tumors, elevated S14 protein was reported to associate with a poor outcome for patients with invasive breast cancer [16]. Furthermore, reduced breast tumor expression of S14, but not FASN, was accompanied by a lower proliferation index in women treated with conjugated linolenic acid [17]. Despite these studies, little consideration has been given to proteins, like S14, that can enhance FASN activity. Although elevated FASN expression and its activity are features of both the differentiated and the neoplastic mammary epithelium, the timing of FASN activation and its cellular context are critical to both the uses of newly synthesized fatty acids and their biological effects upon mammary cells. Our observation that S14 can stimulate FASN enzymatic activity *in vitro* led to the hypothesis that overexpression of S14 in the mammary epithelium would support tumorigenesis through its ability to enhance *de novo* fatty acid biosynthesis. To date, no studies have directly addressed a causal link between S14 activation of *de novo* fatty acid synthesis and mammary tumorigenesis *in vivo*. In the present study, we have modulated the levels of *de novo* synthesized fatty acids in mammary tumors using two transgenic mouse models that either overexpress *Thrsp* in the mammary gland or lack *Thrsp*. Overexpression of *Thrsp* in the mammary glands of MMTV-Neu mice shortened tumor latency and increased tumor proliferation, which was associated with increased levels of MCFA (carbon chain ≤16). Conversely, *Thrsp* loss from MMTV-PyMT mice resulted in slower tumor growth, reduced FASN activity, and lower levels of MCFA compared to controls. MMTV-PyMT tumors lacking *Thrsp* also had reduced levels of phosphorylated Src and Akt, suggesting a possible role for MCFA in potentiating signaling pathways in tumor cells. Surprisingly, tumors from MMTV-Neu mice overexpressing *Thrsp* had a significant increase the expression of genes associated with luminal differentiation, which was concomitant with decreased lung metastasis. Collectively, these data demonstrate a role for S14 in regulating fatty acid synthesis and mammary tumorigenesis *in vivo*.

## Methods

### Mice

The MMTV-Neu line 202 mice [18] (FVB/N-Tg(MMTVneu)202Mul/J; stock number 002376) and the MMTV-PyMT [19] (FVB/N-Tg(MMTVPyVT)634Mul/J; stock number 002374) were obtained from The Jackson Laboratory (Bar Harbor, MN, USA). The *Thrsp* -/- mice [3], herein referred to as S14 -/-, (C57Bl/6 background) were kindly provided by Cary Mariash (IU Health Hospital, Indianapolis, IN, USA). S14 -/- mice were backcrossed for ten generations to FVB/N mice using a speed congenic approach. All mice were genotyped by PCR amplification of DNA isolated from tail tissue. The MMTV-S14 mice have been described previously [4]. Hemizygous MMTV-Neu males were crossed to hemizygous MMTV-S14 females and the resulting offspring genotyped for the presence of both transgenes. Female offspring carrying the *Neu* transgene or both the *Neu* and S14 transgenes were used for tumor studies. Mice were sacrificed when the largest tumor reached 0.5 cm^3^.

Male MMTV-PyMT hemizygous mice were crossed with female S14^−/−^ mice and the progeny genotyped for the presence of the MMTV-PyMT transgene. The resulting MMTV-PyMT, S14^+/−^ male mice were crossed with S14^+/−^ females. The offspring used for the tumor studies were MMTV-PyMT/S14^−/−^ and MMTV-PyMT/ S14^+/+^ females. MMTV-PyMT/Spot14^+/−^ mice were not analyzed in this study. For brevity, the MMTV-PyMT mice are referred to as PyMT and the MMTV-PyMT/S14^−/−^ mice are referred to as PyMT/S14^−/−^ in the text. All animals were maintained in the Center for Comparative Medicine and were provided water and standard rodent chow *ad libitum*. These studies were approved by the University of Colorado Denver Institutional Animal Care and Use Committee.

### Histological analyses

For immunohistochemistry (IHC), dissected tumors were fixed in 10% neutral buffer formalin (NBF) and processed by the Pathology Shared Resource of the University of Colorado Cancer Center. Five-micrometer sections were processed for standard staining with hematoxylin and eosin. For IHC analysis of Ki67, antigen retrieval was performed in a pressure cooker using Dako Tissue Retrieval Solution, pH 6 (Dako, Carpinteria, CA, USA; S1699). Following incubation with 3% H_2_O_2_ (Sigma Aldrich, St. Louis, MO, USA)) in methanol, slides were blocked with Dako Protein Block (Dako, Carpinteria, CA, USA; X0909) and incubated overnight at 4C with rat anti-Ki67 antibody (Dako Carpinteria, CA, USA; M7249) diluted in Dako antibody diluent (Dako, Carpinteria, CA, USA; S0809). The ImmPRESS anti-rat Ig (Vector Labs, Burlingame, CA, USA; MP7444) was used at room temperature for 1 hour and slides were developed using the Dako DAB chromagen kit (Dako, Carpinteria, CA, USA; K3468). Slides were counterstained with Mayer’s hematoxylin. Ki67 staining was quantified using the Ventana Medical Systems (Tucson, AZ, USA) VIAS camera and software. Five to twelve non-overlapping 20x fields in five tissue sections of each group were used to determine the percent of Ki67-positive cells.

For mammary whole mounts, inguinal mammary glands were dissected, fixed in 10% NBF overnight, stained with carmine alum solution, dehydrated, and mounted between glass slides for visualization. A detailed description has been published elsewhere [20].

### Gas chromatography-mass spectrometry (GC-MS) analysis

Tumor fatty acids were isolated and analyzed by GC-MS as previously described [4].

### FASN activity assay

Tumors were pulverized in liquid nitrogen and 30 to 60 mg of tissue was lysed in FASN activity buffer to generate cytosolic lysates (250 mM sucrose, 20 mM HEPES pH 7.6, 2 mM MgCl_2_, 1 mM DTT, 1 mM EDTA, 50 mM NaF, 5 mM NaVO_4,_ and 3x final concentration of Halt™ Protease Inhibitor cocktail (Thermo Scientific; Waltham, MA, USA)). FASN abundance was quantified for each sample and a volume of cytosolic lysate representing 5 μg of FASN per sample was analyzed for FASN activity as previously described [4],[21].

### Immunoblot analysis

Tumors were pulverized in liquid nitrogen and protein was extracted from a portion of the tissue by homogenizing in cell lysis buffer (50 mM Tris, pH 7.4, 150 mM NaCl, 2 mM EDTA, 50 mM NaF, 1% Trition X-100, 1% DOC, 0.1% SDS, 1 mM DTT, 5 mM sodium orthovanadate) containing protease inhibitor cocktail (10 μg/μl leupeptin, 10 μg/μl aprotinin, 10 μM pepstatin A, 1 mM PMSF (L2023, A1153, P4265, and P7626, respectively; Sigma Aldrich, St. Louis, MO, USA)). The homogenates were centrifuged at 13,900 rpm for 15 minutes at 4°C, and supernatants were collected, snap frozen and stored at −20°C until use. Anti-phospho-Akt Ser473 (#9271), anti-Akt (#9272), anti-phospho-Src Tyr416 (#2101), anti-phospho-PI3K p85 (#4292), anti-PI3K p85 (#4257), anti-ACLY (#4332), anti-ACC (#3676), and anti-Actin (#5125) were obtained from Cell Signaling Technology (Beverly, MA, USA). Anti-PyMT (sc-53481), anti-Neu (sc-284), anti-FASN (sc-20140), anti-β-tubulin (sc-9104), and anti-Krt18 (sc-28264) were obtained from Santa Cruz Biotechnology (Santa Cruz, CA, USA). The anti-S14 antibody (ab97504) was obtained from Abcam (Cambridge, MA, USA). The monoclonal antibody directed against amino acids 2 to 17 of Src was prepared by the University of Colorado Cancer Center Monoclonal Antibody, Protein Production, and Tissue Culture Shared Resource. Protein bands were visualized using SuperSignal West Pico chemiluminescent substrate (Thermo Scientific, Waltham, MA, USA) and film. ImageJ [22] was used to quantify protein bands. Alternatively, cytosolic extracts were prepared as described in the FASN Activity Assay section, analyzed by SDS-PAGE and imaged/quantified using the LiCOR Odyssey system as described previously [4].

### Quantitative PCR (qPCR)

Gene expression was analyzed by qPCR using primers and FAM-labeled probes or SYBR green chemistry. Primers and probe sequences or catalog numbers are listed in Additional file 1. Unless otherwise indicated, all genes assayed are mouse. Transcript copy number was estimated using a standard curve generated from a known concentration of amplicon for each reaction. Additional details have been described elsewhere [23].

### Cell culture

Human breast cancer cells were provided by the University of Colorado Cancer Center Protein Production, Monoclonal Antibody and Tissue Culture Shared Resource and used within 6 months. Cell line identity was validated by STR analysis prior to purchase. Cells were grown in DMEM (Sigma Aldrich, St. Louis, MO, USA) supplemented with 10% fetal bovine serum (Hyclone, Thermo Scientific, Waltham, MA, USA) and 1% antibiotic/anti-mycotic solution (Sigma Aldrich, St. Louis, MO, USA). RNA was isolated using the RNEasy Mini Kit (Qiagen, Valencia, CA, USA) and cDNA was prepared using the Verso cDNA synthesis kit (Thermo Scientific, Waltham, MA, USA).

### Human microarray data analysis

Datasets of primary human breast tumors were obtained from the NCBI Gene Expression Omnibus (GEO) or from Oncomine [24]. All data were normalized within datasets. Datasets were individually sorted by S14 expression, and the top and bottom 25% of the samples were categorized as high and low S14-expressing, respectively. Details of each dataset are listed in Additional file 2. Within the high and low S14-expressing groups, those samples for which either estrogen receptor (ER) IHC status (positive or negative) and intrinsic subtype classification (luminal or basal) were available were used to create 2 × 2 contingency tables. Fisher’s exact test was used to determine statistical significance for comparisons of tumor subtypes and ER status.

### Tumor microarray analysis

Total RNA was isolated from tumor tissue using Trizol (Life Technologies, Carlsbad, CA, USA). The RNEasy Mini Kit (Qiagen, Valencia, CA, USA) with optional on-column DNAse digestion was used on the final RNA. One hundred micrograms (Neu and Neu/S14 tumors) or 250 micrograms (PyMT and PyMT/S14^−/−^ tumors) of total RNA was prepared for microarray analysis using the Whole Transcript Expression RNA kit (Life Technologies, Carlsbad, CA, USA) and samples were hybridized to mouse 1.1 ST gene arrays (Affymetrix, Santa Clara, CA, USA). Arrays were washed, stained and imaged using the Affymetrix Gene Atlas Microarray System. Raw data were analyzed using Partek Genomic Suite. Select genes were validated by qPCR (see Additional file 3). These studies can be accessed the NCBI GEO database [GSE42708 and GSE55886]. GO enrichment analysis was performed using the Database for Annotation, Visualization and Integrated Discovery (DAVID) bioinformatics resource [25],[26]. Only genes with fold change of +/− 1.2 and *P* <0.05 were considered for analyses, and separate DAVID analyses were performed on genes that were either increased or decreased to allow an interpretation of networks that were enriched in either direction. Full gene lists are available upon request. We presented those GO terms that were enriched ≥2-fold with *P* ≤0.01. Four tumors from PyMT and PyMT, S14^−/−^ mice were analyzed, thirteen tumors from Neu mice, and fourteen tumors from Neu/S14mice.

### Statistical analysis

Unless otherwise stated, the unpaired, two-tailed *t*-test was performed to determine statistical significance.

## Results

**Figure 1:**
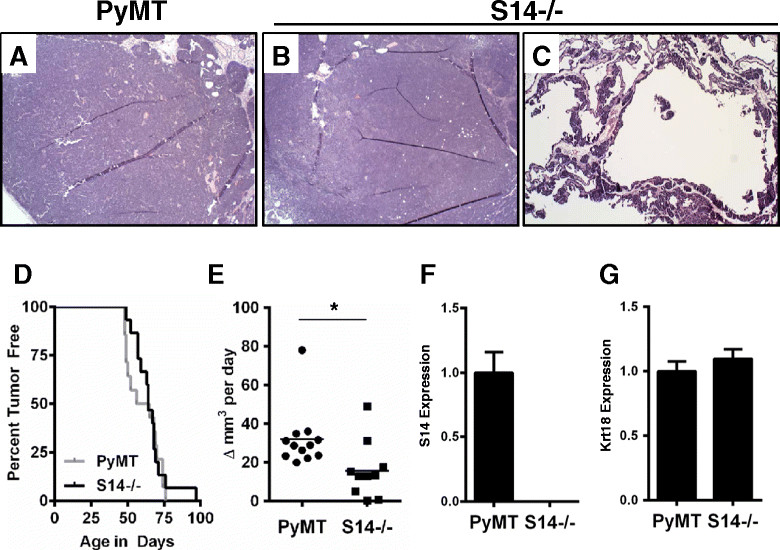
**Tumor histology, latency, and growth in polyomavirus middle T antigen (PyMT) mice with and without S14.** H&E-stained sections of tumors from PyMT **(A)**, and PyMT/S14^−/−^ (S14^−/−^) mice **(B**, **C)**. A representative solid tumor is shown in **(B)**. A tumor with a cystic morphology is shown in **(C)**. Images were captured at 10x magnification. **(D)** Kaplan Meier survival curve; tumors appeared with median latencies of 61 days (PyMT; n = 15) and 64 days (S14^−/−^; n = 14); hazard ratio (HR) 1.187 (0.588 to 2.55); log rank *P*-value = 0.62. **(E)** Tumor growth rates; PyMT n = 12, S14^−/−^ n = 10; *P = 0.02. **(F)** Expression of S14 (THRSP) and **(G)** keratin 18 (Krt18) in tumors from S14^−/−^ (n = 13) relative to PyMT (n = 14) mice. Graphs are mean + standard error of the mean.

We used MMTV-PyMT mice to study the effects of S14 loss because they rapidly develop mammary tumors at a young age, providing an opportunity to observe any delay in tumor latency in mice with altered mammary *de novo* fatty acid synthesis. Based on the reported link between decreased S14 expression and reduced tumor cell proliferation in human breast cancer [17], we hypothesized that loss of S14 would delay mammary tumor latency. All PyMT mice, and approximately 50% of PyMT/S14^−/−^ mice in our study formed multiple, large, solid tumors (Figure 1A and B). However, in the remaining PyMT/S14^−/−^ mice, we observed large cystic structures (Figure 1C). Once this tumor phenotype emerged, the cysts filled rapidly with fluid, such that the volume of the tumor expanded to 500 mm^3^ in less than a week (data not shown). The median time to palpable tumor formation in PyMT control female mice was 61 days, while the median time to palpable tumor formation in PyMT/S14^−/−^ female mice was 64 days (Figure 1D). While loss of S14 did not delay the formation of palpable tumors, the solid tumors lacking S14 grew significantly slower than PyMT tumors (Figure 1E). S14 mRNA levels in tumors were evaluated by qPCR (Figure 1F) and expression of *Krt18* was examined as a control (Figure 1G).

**Figure 2:**
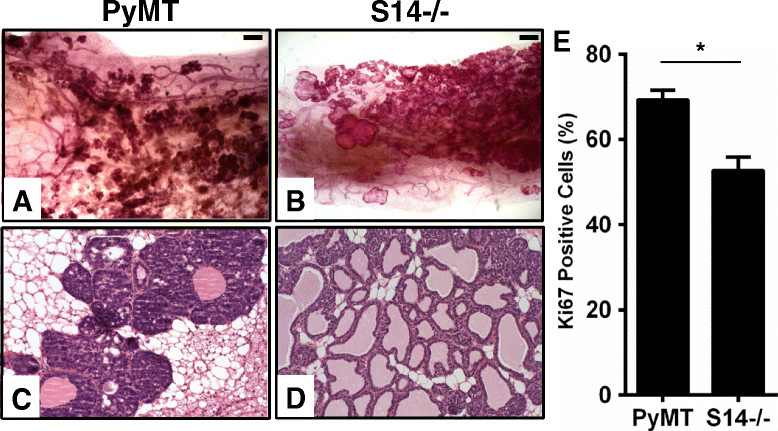
**Mammary gland morphology and proliferation in polyomavirus middle T antigen (PyMT) and PyMT/S14** ^**−/−**^**mice.** Whole mounted mammary glands from 10-week-old PyMT **(A)** and PyMT/S14^−/−^ (S14^−/−^) **(B)** female mice. Scale is 500 μm. H&E-stained sections of mammary glands from PyMT **(C)** and S14^−/−^**(D)** mice, captured at 20x magnification. **(E)** Quantification of Ki67 immunohistochemistry (number of positive nuclei out of total) in mammary glands from 10-week-old mice; n = 5 mice per group; graph is mean + standard error of the mean; *P* <0.005.

Mammary glands were collected from 10-week-old PyMT and PyMT/S14^−/−^ females and evaluated as whole mounts, to examine the structure of the ductal tree. Interestingly, we observed striking differences in the mammary gland structures between PyMT and PyMT/S14^−/−^ mice at this age. As previously reported [19], PyMT mice initially developed mammary tumors near the nipple region, while some hyperplastic structures were observed in the middle and at the distal ends of the mammary tree (Figure 2A). By the time PyMT animals were 10 weeks old, the majority of the mammary gland was filled with hyperplastic epithelial structures and very few normal ducts remained (Figure 2A and C). In contrast, PyMT/S14^−/−^ mice formed large, dilated, fluid-filled structures and we observed few solid hyperplasias in 10-week old females (Figure 2B and D). Cell proliferation throughout the gland in PyMT/S14^−/−^ mice was significantly decreased relative to PyMT controls (Figure 2E), while no differences were observed in apoptotic cells between PyMT and PyMT/S14^−/−^ mice at any age (data not shown).

**Figure 3:**
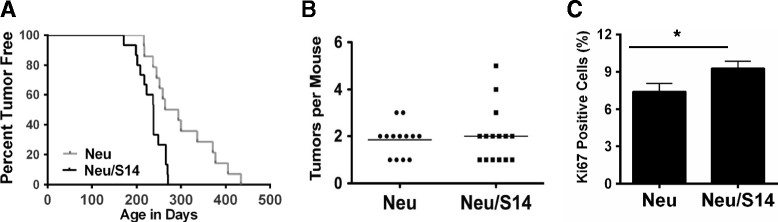
**Tumor latency, multiplicity, and proliferation in Neu and Neu/S14 mice. (A)** Kaplan-Meier survival curve of tumor latency in Neu and Neu/S14 mice. Median times to tumor onset were 238 days (Neu/S14) and 279 days (Neu); hazard ratio (HR) 0.2645 (0.1087 to 0.6435); log rank *P*-value = 0.0034. **(B)** Number of tumors per mouse at sacrifice. *P* = 0.52. **(C)** Quantification of Ki67 immunohistochemistry (number of positive nuclei out of total) in tumors; n = 5 mice per group; graph is mean + standard error of the mean; *P* = 0.02.

The effects of S14 overexpression on tumorigenesis were determined using MMTV-Spot14 transgenic female mice crossed with male MMTV-Neu mice. Female MMTV-Neu mice develop tumors with a longer latency than MMTV-PyMT mice, with the median time to tumor formation reported to be more than 200 days [18]. Consistent with our hypothesis, overexpression of S14 in MMTV-Neu mice significantly shortened tumor latency (Figure 3A), with median times to palpable tumor formation of 238 days in Neu/S14 mice and 279 days in Neu mice. Most Neu mice developed one or two tumors per animal, and the number of tumors detected per mouse was not affected by S14 overexpression (Figure 3B). Tumor cell proliferation was modestly but significantly increased in Neu/S14 compared to Neu mice (Figure 3C). Overexpression of S14 was confirmed by immunoblot analysis (Additional file 4A and B) and by qPCR (Additional file 4C) and did not alter the protein or gene expression levels of Neu in mammary tumors (Additional file 4D and E).

**Figure 4:**
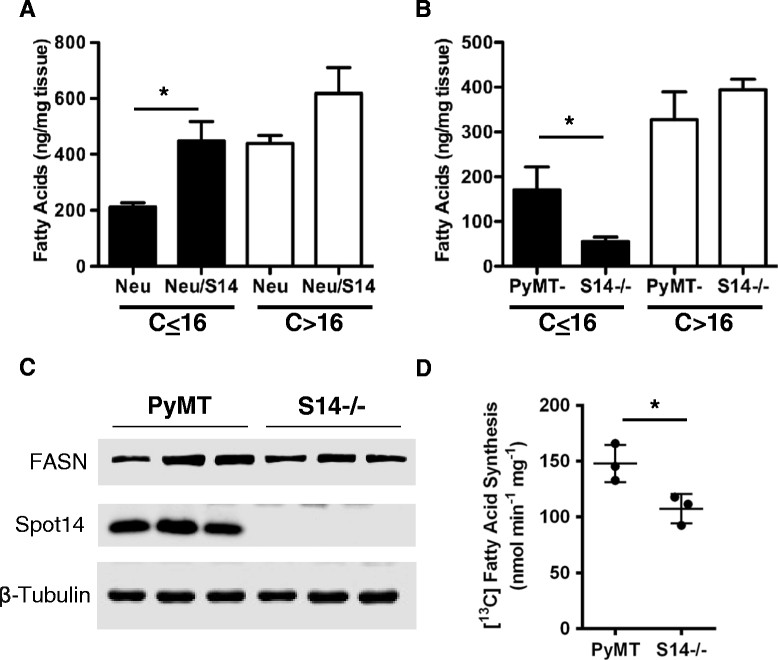
**Gas chromatography-mass spectrometry (GCMS) analysis of tumor fatty acids. (A)** Tumor fatty acid contents from Neu (n = 8) and Neu/S14 (n = 7) mice. *P* ≤0.05. **(B)** Tumor fatty acid contents from polyomavirus middle T antigen (PyMT) (n = 6) and S14^−/−^ (S14^−/−^) (n = 6) mice. Fatty acids are grouped according to chain length. Those with carbon chains ≤16 are on the left of each graph, and those with carbon chains >16 are on the right of each graph. **(C)** Immunoblot analysis of proteins in cytosolic lysates used for fatty acid synthase (FASN) activity assay showing FASN (top) and S14 (middle). β-Tubulin was used as a loading control. **(D)** FASN activity in cytosolic lysates from PyMT or S14^−/−^ tumors. *P* = 0.035. Graphs are mean + standard error of the mean.

We have recently shown that addition of S14 to recombinant FASN enhances the yield of MCFA *in vitro*, and S14 overexpression in the mammary epithelium increased the amount of MCFA present in milk during lactation [4]. GCMS was used to evaluate the fatty acid profile of tumors from Neu versus Neu/S14 mice and from PyMT versus PyMT/S14^−/−^ (Additional file 5). We observed increased levels of many fatty acids when comparing Neu/S14 to Neu tumors. The largest increase in tumor fatty acids was seen in the MCFA group (chain lengths ≤16 carbons), and there were no significant differences in fatty acids >16 carbons (Figure 4A). Conversely, in PyMT/S14^−/−^ tumors, MCFA were significantly lower compared to PyMT tumors, and significant differences were not seen in fatty acids >16 carbons (Figure 4B). Analysis of cytosolic lysates revealed that PyMT tumors lacking S14 had equivalent levels of FASN protein as PyMT control tumors (Figure 4C) but had significantly lower FASN enzyme activity (Figure 4D), suggesting that the decreased levels of MCFA in the PyMT/S14^−/−^ model resulted from reduced *de novo* fatty acid synthesis. We did not detect any quantitative differences in gene or protein levels of ACLY, ACC, or FASN resulting from S14 loss of S14 overexpression *in vivo* (Additional file 6). In summary, these data indicate that the effects of S14 on the level of MCFA in tumors may result from its ability to modulate the enzymatic activity of FASN.

**Figure 5:**
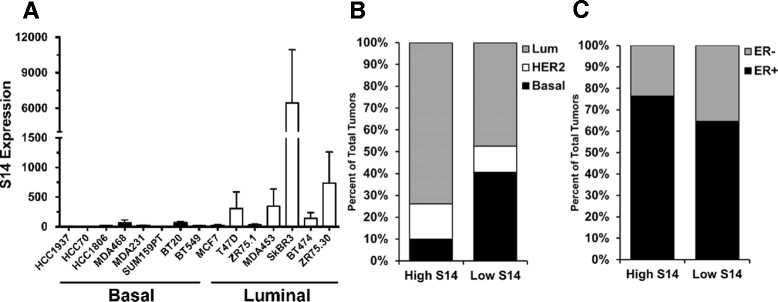
**Association between S14 and tumor differentiation markers. (A)** S14 expression in human breast cancer cells, normalized to actin expression. Graph is mean + standard error of the mean. **(B)** Tumors with high S14 (n = 192) are more likely to be luminal versus basal (*P* = 0.0002) compared to tumors with low S14 (n = 204). **(C)** Tumors with high S14 (n = 436) are more likely to be estrogen receptor (ER) + versus ER− (*P* <0.0001) than tumors with low S14 (n = 438).

Lung tissue was collected from all mice in both the PyMT/S14^−/−^ and the Neu/S14 mice and their respective controls, to examine for metastases. No significant differences were found in either the number of mice with lung metastases or in the number of lesions per lung between PyMT and PyMT/S14^−/−^ mice (Figure 5A and B), suggesting that the loss of S14, and associated decrease in the proliferation of primary tumor cells, did not impair the process of metastasis in the PyMT model. In contrast, overexpression of S14 in the Neu model resulted in a surprising decrease of lung metastases. Only one of the 15 Neu/S14 mice examined had visible lung metastases. In comparison, 6/17 Neu mice had metastatic lesions in their lungs (Figure 5C). Representative lung lesions from both mouse models are shown in Figure 5 (5D-5G). A close examination of primary tumors indicated evidence of lympho-vascular invasion (LVI) in 3/17 Neu mice (data not shown), while no LVI was observed in primary tumors from Neu/S14 mice.

Microarray analysis was performed to better characterize the differences between tumors from PyMT and PyMT/S14^−/−^ mice and also between tumors from Neu and Neu/S14 mice. After identifying genes that were significantly increased or decreased between different experimental groups (PyMT versus PyMT/S14^−/−^ or Neu versus Neu/S14), we performed gene ontology (GO) enrichment analyses to identify the biological processes that were impacted by S14 manipulation.

In PyMT tumors, the loss of S14 led to reduced expression of genes associated with glucose metabolism, protein kinase activity, and cell proliferation (Additional file 7). In this model, the only significantly enriched GO term for genes that increased in tumors lacking S14 was the cell cycle (fold enrichment = 2.81; *P* = 0.002). S14 overexpression in Neu tumors was associated with elevated levels of genes involved in protein translation, positive regulation of growth, and the electron transport chain (Additional file 8). Conversely, tumors overexpressing S14 had decreased levels of genes associated with the development of the heart, skeletal system, and blood vessels (Additional file 9). The genes in these networks appeared to generally represent two categories with regards to the mammary gland and breast cancer. They have been reported to function during early mammary placode formation in the developing embryo (that is, *Msx2*, *Tbx3*, *Wnt3a*, and *Fgfr1*) or they are involved in breast cancer cell migration, invasion, and proliferation (that is, *Barx2*, *Pdgfrb*, *Bmp6*, and *Jak3*) [27]-[36].

When taken together, these data have several implications. First, we observe differential effects of S14 loss or overexpression on metabolic pathways. Loss of S14 from PyMT tumors is linked to decreased glycolytic gene expression, while S14 overexpression in Neu tumors is associated with increased expression of electron transport chain genes. Because PyMT-driven tumors grow so much more rapidly than those in Neu mice, we postulate that tumors from PyMT mice are highly dependent on glycolysis for ATP production and to generate building blocks for cell division. The observation that S14 loss from PyMT tumors leads to a decrease in cell proliferation is not surprising given that these tumors also have a decrease in glycolytic genes, as cell cycle progression and metabolism are tightly intertwined. Conversely, because S14 overexpression in Neu tumors is associated with elevated proliferation, those tumors are also likely more metabolically active, and this is reflected in greater expression of mitochondrial metabolism genes. Overall, the gene expression data are consistent with the differences in tumor growth rates and cell proliferation resulting from S14 loss or overexpression in both mouse models.

In addition to those related to metabolism, we observed differences in genes known to be associated with functional differentiation of the normal mammary gland in Neu/S14 tumors (Figure 5H). One of the most significantly elevated genes in Neu/S14 tumors was ETS-related transcription factor *Elf5*, which regulates alveolar differentiation during pregnancy in the mouse mammary gland [37]. The expression of milk protein genes *Csn2*, *Csn1s1*, and *Csn1s2a* was also elevated in Neu/S14 versus Neu tumors (Figure 5H). Recently, Elf5 was shown to repress an EMT-like phenotype during normal mammary gland development and to prevent metastasis in two mouse models of mammary tumorigenesis [38]. When we consider the overall gene expression profile of tumors from Neu/S14 mice, we postulate that overexpression of S14 is associated with reduced levels of genes associated with early mammary stem cells and with breast cancer invasion and metastasis, as well as the elevated expression of genes involved with alveolar epithelial differentiation. Altogether, this gene expression profile is consistent with reduced tumor metastasis, associated with S14 overexpression, observed in this model (Figure 5C). Expression of *Csn* genes was also evaluated in the tumors from PyMT and PyMT/S14^−/−^ mice (Figure 5I). There was no significant difference in the expression of *Csn2* and *Csn1s2a* between PyMT tumors and the solid tumors from PyMT/S14^−/−^ mice (Figure 5I); however, the cystic tumors that formed in PyMT/S14^−/−^ mice had lower expression of *Csn2* and *Csn1s2a* than those from PyMT mice or than the solid tumors from PyMT/S14^−/−^ mice, indicating an altered differentiation profile in a subset of PyMT/S14^−/−^ tumors. *Krt18* expression was not different between any tumor groups (Figure 5I).

**Figure 6:**
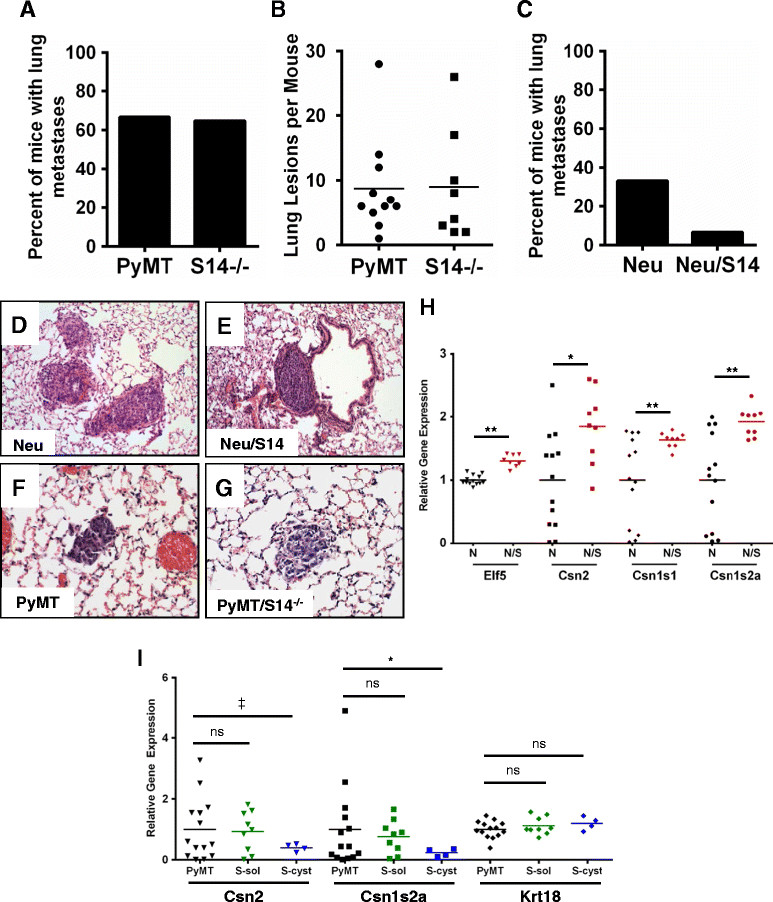
**Influence of S14 on tumor metastasis and gene expression. (A)** Percent of mice with visible lung metastases upon H&E examination in polyomavirus middle T antigen (PyMT) (11/17) or PyMT/S14^−/−^ (S14^−/−^; 8/12) mice; Fisher’s exact *P*-value = 1.0. **(B)** Number of visible lung lesions in PyMT or S14^−/−^ mice. Fisher’s exact *P*-value = 0.9. **(C)** Percent of mice with visible lung metastases upon H&E examination in Neu (6/17) or Neu/S14 (1/15) mice. Fisher’s exact *P*-value = 0.09. Representative metastatic lesions in lungs from Neu **(D)**, Neu/S14 **(E)**, PyMT **(F)** and S14^−/−^**(G)** mice. **(D)** and **(E)** were captured at 20x magnification; **(F)** and **(G)** were captured at 40x magnification. **(H)** Expression of genes associated with mammary epithelial cell differentiation in primary tumors from Neu (N; black) and Neu/S14 (N/S; red) mice. **(I)** Csn2, Csn1s2a, and Krt18 expression in tumors from PyMT and PyMT/S14^−/−^ mice. Tumors from PyMT/S14^−/−^ (S) mice are categorized as solid (S-sol; green) or cystic (S-cyst; blue). ^‡^*P* <0.1; *P <0.05; **P ≤0.01; ns = not significant.

In human breast cancer cell lines, S14 expression was highest in those representing the luminal subtype compared to those cell lines with basal characteristics (Figure 6A). To determine whether S14 is associated with a particular subtype of human breast tumors, we analyzed publicly available microarray data. Tumors with high S14 expression were significantly more likely to be luminal than basal, when considering the intrinsic subtype [39], and were also significantly more likely to be ER+ than ER− (Figure 6B and C). Because the gene expression profile of the Neu/S14 tumors was reminiscent of the differentiated mammary gland, we examined the non-tumor bearing mammary glands from age-matched (10 month old), and diestrus-staged Neu and Neu/S14 females. Compared to Neu mice, glands from Neu/S14 mice had denser epithelium and apparent hyperplastic epithelial structures (Additional file 10). Further investigation into the connection between S14 and features of luminal differentiation in human breast tumors is warranted.

**Figure 7:**
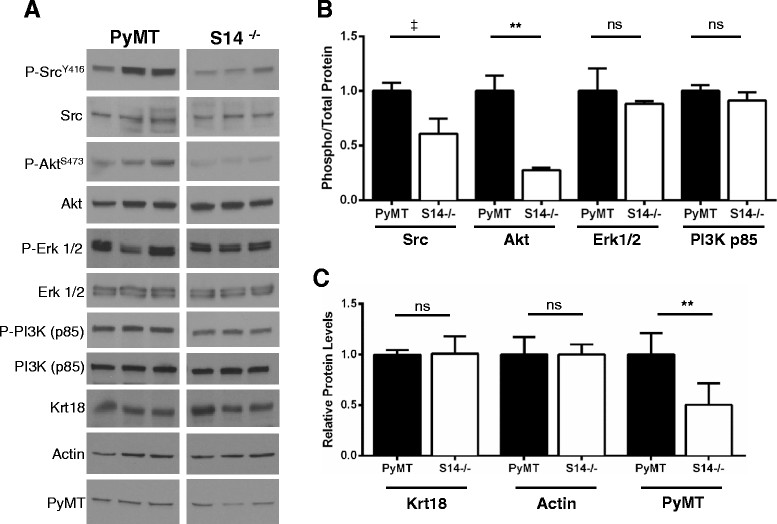
**Immunoblot analysis of solid tumors from polyomavirus middle T antigen (PyMT) and PyMT/S14** ^**−/−**^**mice. (A)** Immunoblots of solid tumors from three PyMT and three PyMT/S14^−/−^ (S14^−/−^) mice. **(B)** Ratios of phosphorylated protein to the total levels of the same protein expressed as levels in S14^−/−^ tumors (white bars) relative to PyMT tumors (gray bars). **(C)** Relative levels of keratin 18 (Krt18), Actin, and PyMT in tumors from S14^−/−^ (white bars) compared to PyMT (gray bars) mice. ^‡^*P* <0.1; **P <0.01; ns = not significant. Graphs are mean + standard error of the mean.

Growth of PyMT tumors is highly dependent on signaling through Src and the PI3K/Akt pathways [40]. When solid tumors from PyMT mice were compared to PyMT/S14^−/−^ solid tumors, the phosphorylation of Src and Akt was significantly decreased in solid tumors lacking S14 (Figure 7A and B). Two out of three PyMT tumors appeared to have higher levels of phosphorylated Erk1/2 than PyMT/S14^−/−^ tumors, but these differences were not significant when the average ratios of phosphorylated to total Erk1/2 of each group were compared. No differences were observed in the phosphorylation status of the p85 subunit of PI3’ kinase (Figure 7A and B). PyMT was expressed in all tumors examined (Figure 7A); however, the levels of PyMT were significantly lower in tumors lacking S14 (Figure 7C). While PyMT does not have any kinase activity, it associates with protein kinases at the cell membrane, and the PyMT protein itself is known to interact with lipid membranes. Little is known about what factors regulate the degradation or turnover of PyMT. The observed differences in Src and Akt phosphorylation could be due to reduced PyMT levels in tumors lacking S14, and changes in all three molecules (Src, Akt, and PyMT) could reflect the alterations in tumor fatty acids, although we did not specifically examine the phospholipid fraction. The levels of actin and Krt18 were equivalent between groups (Figure 7A and C).

## Discussion

In this study, the role of S14 in the regulation of mammary tumor growth was investigated *in vivo* using mouse models in which S14 levels were genetically modified. We and others have previously shown that S14 regulates *de novo* fatty acid synthesis in the normal mammary epithelium during lactation [3],[4]; and others have shown that S14 regulates *de novo* fatty acid synthesis in breast cancer cell lines [6],[41],[42]. We recently showed that S14 altered FASN enzyme catalysis *in vitro* by increasing the apparent V_max_ and decreasing the apparent Km, which resulted in a S14-mediated enhancement of *de novo* fatty acid synthesis [4]. Furthermore, we showed that S14 transgenic mice produced significantly greater quantities of MCFA in milk during lactation [4]. Here, we have demonstrated a role for S14 in modulating the biology of mammary tumors, potentially through modulation of *de novo* fatty acid synthesis. Overexpression of S14 in Neu-induced mammary tumors shortened tumor latency and increased tumor cell proliferation (Figure 3); and these differences in tumor growth were associated with elevated tumor levels of MCFA relative to Neu controls (Figure 4). Conversely, the absence of S14 from PyMT-induced mammary tumors was associated with slower tumor growth and decreased proliferation (Figures 1 and 2), which was coincident with decreased FASN activity and lower levels of MCFA in tumors lacking S14 compared to controls (Figure 4). Combined, both tumor models suggest that S14 potentiates *de novo* fatty acid synthesis *in vivo*, which could result in altered mammary tumor growth.

Analysis of human breast tumors identified an association between high S14 expression and features of luminal differentiation (Figure 6). Specifically, tumors with high S14 expression were significantly more likely to represent the luminal versus basal subtype, and were more likely to be ER+ than ER−. Consistent with the observation from human breast tumors, tumors from Neu/S14 mice also had increased expression of many genes associated with luminal differentiation of mammary epithelial cells, compared to those from Neu mice. Contrary to our hypothesis, overexpression of S14 in Neu-induced tumors resulted in fewer lung metastases compared to controls. These results suggest that tumors from Neu/S14 mice may be less likely to metastasize, in part, because the cells are well differentiated. We have found that overexpression of S14 in normal mouse mammary epithelial cells did not increase the expression of genes associated with lactogenic differentiation (data not shown). Moreover, MMTV-S14 transgenic mice do not display features of lactogenic differentiation in either the gene expression profiles or gland morphology [4]. The frequency of mammary tumor metastasis was also quantified in the PyMT mice with or without S14. While loss of S14 expression was associated with reduced tumor cell proliferation, it did not appear to affect tumor metastasis to the lungs in PyMT mice (Figure 6), suggesting that the effects of S14 on tumor cell proliferation could be independent of processes that govern tumor metastasis.

S14 (reported as *Thrsp*), one of several genes associated with luminal epithelial differentiation, was found to be downregulated as PyMT-driven mammary tumors progressed from adenomas to carcinomas [43], indicating that S14 may be expressed in a relatively well-differentiated tumor cell type in the PyMT model. Notably, in the PyMT model, only mice lacking S14 expression produced cystic lesions, while PyMT mice formed solid tumors, and the cystic lesions appearing in PyMT/S14^−/−^ mice had reduced expression of casein genes compared to the solid lesions from either group of mice (Figure 5). This observation is consistent with the observed gene expression profile in tumors from Neu/S14 mice [43]. The cystic lesions that developed in the absence of S14 were reminiscent of those that formed in the same model when the mice were treated with the Src inhibitor SKI606 [44]; however, Hebbard *et al*. reported expression of β-Casein in the lumens of the lesions from SKI606-treated mice. Although the lesions that form in PyMT mice treated with the Src inhibitor and those from PyMT/S14^−/−^ mice appear similar in morphology, they likely do not have the same molecular characteristics with regards to cellular differentiation. Cumulatively, these data indicate that S14 itself is not sufficient to promote a differentiation program in mammary tumors; rather, S14 may support the proliferation or survival of a differentiated mammary tumor cell type.

Both Src and Akt rely on cellular lipids for their activity [45]-[47]. Src-family kinases are modified by myristate and palmitate, both of which are MCFA, and myristoylation of c-Src enhances its kinase activity [45]. The lipid-dependent activation of Akt occurs through the binding of its plextrin-homology (PH) domain to phosphatidyl-inositol-trisphosphate (PIP_3_) present in the plasma membrane [46],[47]. The phosphorylation of Src and Akt at their active sites was decreased in PyMT tumors lacking S14 (Figure 7), suggesting that signal transduction through these kinases may be diminished; however, we did not examine the in vivo activities of these enzymes. In addition, levels of PyMT were reduced in tumors lacking S14. Currently, it is unclear how modulating S14 levels or *de novo* fatty acid synthesis affects the membrane lipid profiles in either PyMT- or Neu-induced mammary tumors. Furthermore, it is not clear how changes in tumor cell lipid profiles impact the post-translational modification or activation of critical signaling molecules, or how changes in membrane phospholipids affect the stability of the PyMT oncogene. Our results suggest that changing the tumor fatty acid profile may be a way to alter pro-tumorigenic signaling pathways and reduce the growth and survival of breast cancer cells. Indeed, several studies have reported direct relationships between expression and activity of FASN and HER2 signaling pathway activation, which notably affected Akt and Erk activity in cancer cells [48]-[50].

Chemical inhibition of *de novo* fatty acid synthesis decreases the proliferation of cultured breast cancer cells, reduces activation of signaling pathways, and in some cases, slows the growth of xenograft tumors [10],[48],[51]-[53]; and direct inhibition of FASN has been explored for cancer therapy [48],[53]-[57]. Clearly, modulating fatty acid synthesis activity alters tumor biology. Our study raises several important questions. Are there unique roles for MCFA versus long chain fatty acids in intracellular signaling and tumor cell proliferation? What role do dietary fatty acids play in modulating the growth of mammary tumors that have reduced FASN activity? This work highlights the potential of studying molecules that influence FASN activity as contributors to tumorigenesis and as potential therapeutic targets for the treatment of breast cancer. Furthermore, it suggests FASN activity need not be completely inhibited; instead, novel therapeutics that influence the spectrum of products produced by FASN may be sufficient to alter tumor properties such as signaling pathway activation, cell proliferation, and metastasis.

## Conclusions

S14 enhances FASN catalysis *in vitro* and the S14 overexpression in the mammary epithelium during lactation facilitated the synthesis of MCFA [4]. Here, we have demonstrated an *in vivo* role for S14 in modulating *de nov*o fatty acid synthesis in two genetic mouse mammary tumor models. Our finding that altered levels of tumor MCFA are associated with changes in tumor cell proliferation, tumor metastasis, and intracellular signal transduction pathway activation of Src and Akt highlights the importance of studying those molecules that influence FASN activity as potential targets for the treatment of breast cancer. Finally, the potential relationship between S14 expression and luminal differentiation in both mouse and human tumors warrants further investigation.

## Additional files

## Electronic supplementary material

Additional file 1: QPCR primer/probe sets. Table of gene names and associated primer and probe sequences or Applied Biosystems catalog numbers of commercial primer/probe sets used for qPCR analysis. (PDF 82 KB)

Additional file 2: Public microarray datasets. This file contains the GEO ID (if applicable), the number of tumors with low or high S14 expression, the array platform used, the PubMed ID references for each dataset, and the specific analysis for which each dataset was used. (PDF 29 KB)

Additional file 3: **QPCR validation of microarray data.** This file contains a table showing qPCR results from microarray targets. The mean expression values with standard error of the mean (SEM) for Neu (n = 13) and Neu/S14 (n = 14) and polyomavirus middle T antigen (PyMT) (n = 14) and S14^−/−^ (n = 9) tumors are shown, with fold changes (Neu/S14 versus Neu or S14^−/−^ vs PyMT) and *P*-values. (PDF 34 KB)

Additional file 4: **Transgene levels in Neu and Neu_S14 Tumors.** (**A**) Immunoblot of S14 in tumors from Neu and Neu/S14 mice, with β-Tubulin as a loading control. (**B**) Quantification of S14 protein levels normalized to B-Tubulin; *P* = 0.06. (**C**) QPCR analysis of S14 gene expression in tumors from Neu (n = 13) and Neu/S14 (n = 12) mice. *P* = 0.025. (**D**) Immunoblot analysis of Neu (ErbB2) in tumors from Neu and Neu/S14 mice, with Erk as a loading control. (**E**) QPCR analysis of Neu transgene expression in tumors from Neu (n = 13) and Neu/S14 (n = 12) mice. (PDF 41 KB)

Additional file 5: **Gas chromatography-mass spectrometry (GCMS)** **tumor fatty acids.** This file contains a table of the fatty acids analyzed in tumors from polyomavirus middle T antigen (PyMT) (n = 6), PyMT/S14^−/−^ (n = 6), Neu (n = 8), and Neu/S14 (n = 7) mice. Included are the fatty acid chain lengths and saturation, the measured fatty acids in ng/mg tissue, the ratios of each group to its respective control, and the *P*-values of the differences between groups. (PDF 54 KB)

Additional file 6: **Effects of S14 on fatty acid synthesis enzymes.** Gene expression and protein levels of ATP-citrate lyase (ACLY), acetyl Co-A carboxylase (ACC), and fatty acid synthase (FASN) in Neu (n = 14) and Neu/S14 (n = 15) tumors (A and B, respectively) and polyomavirus middle T antigen (PyMT) (n = 4) and S14^−/−^ (n = 4) tumors (C and D, respectively). Immunoblots were quantified for Neu and Neu/S14 as well as PyMT and S14^−/−^ tumors in E and F, respectively. ns, not significant. (PDF 34 KB)

Additional file 7: **Gene Ontology (GO) enrichment genes DOWN in** **polyomavirus middle T antigen (** **PyMT)_S14null versus PyMT.** This file contains the list of significantly enriched GO (biological process) terms for genes decreased in PyMT/S14^−/−^ vs PyMT tumors, as determined using the Database for Annotation, Visualization and Integrated Discovery (DAVID) resource. (XLSX 11 KB)

Additional file 8: Gene Ontology (GO) enrichment genes UP in Neu_S14 v Neu. This file contains the list of significantly enriched GO (biological process) terms for genes increased in Neu/S14 vs Neu tumors, as determined using the Database for Annotation, Visualization and Integrated Discovery (DAVID) resource. (XLSX 11 KB)

Additional file 9: Gene Ontology (GO) enrichment genes DOWN in Neu_S14 v Neu. This file contains the list of significantly enriched GO (biological process) terms for genes decreased in Neu/S14 vs Neu tumors, as determined using the Database for Annotation, Visualization and Integrated Discovery (DAVID) resource. (XLSX 9 KB)

Additional file 10: MG whole mounts Neu and Neu_S14. This file contains images of whole mounted mammary glands from two each of Neu and Neu/S14 mice at 10 months of age and in diestrus. Scale for left column (2x) is 1 mm, scale for right column (10x) is 200 μm. (PDF 642 KB)

Below are the links to the authors’ original submitted files for images.

Authors’ original file for figure 1

Authors’ original file for figure 2

Authors’ original file for figure 3

Authors’ original file for figure 4

Authors’ original file for figure 5

Authors’ original file for figure 6

Authors’ original file for figure 7

## Abbreviations

ACC: acetyl Co-A carboxylase
ACLY: ATP-citrate lyase
DAVID: Database for Annotation, Visualization and Integrated Discovery
DMEM: Dulbecco's modified Eagle's medium
ER: estrogen receptor
FAM: 6-carboxyfluoroescein
FASN: fatty acid synthase
GCMS: gas chromatography-mass spectrometry
GEO: NCBI Gene Expression Omnibus
GO: Gene Ontology
H&E: hematoxylin and eosin
HR: hazard ratio
IHC: immunohistochemistry
KEGG: Kyoto Encyclopedia of Genes and Genomes
Krt18: Keratin 18
LVI: lympho-vascular invasion
MCFA: medium chain fatty acids
MMTV: mouse mammary tumor virus
PyMT: polyomavirus middle T antigen
S14: Spot 14
THRSP: thyroid hormone responsive protein Spot 14
