# Supplementary figures and images for: Modulation of tumor fatty acids, through overexpression or loss of thyroid hormone responsive protein spot 14 is associated with altered growth and metastasis
Source: Breast Cancer Res. 2014 Dec 4;16:481. doi: 10.1186/s13058-014-0481-z (PMC4303195; doi:10.1186/s13058-014-0481-z)

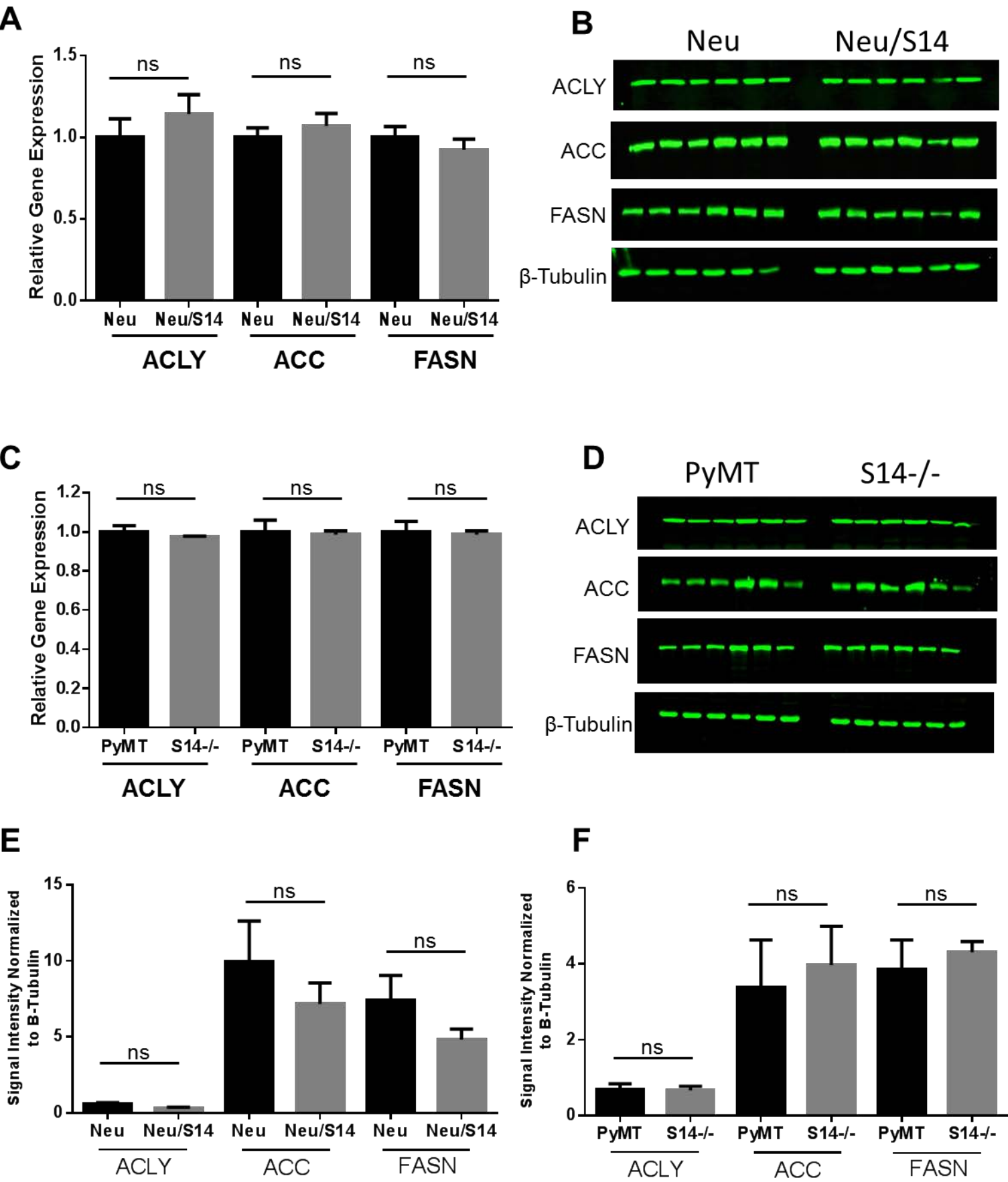

Supplement: Supplementary file 1 — Additional file 1: QPCR primer/probe sets. Table of gene names and associated primer and probe sequences or Applied Biosystems catalog numbers of commercial primer/probe sets used for qPCR analysis. (PDF 82 KB) [file 13058_2014_481_MOESM1_ESM.pdf]

## Additional File 3

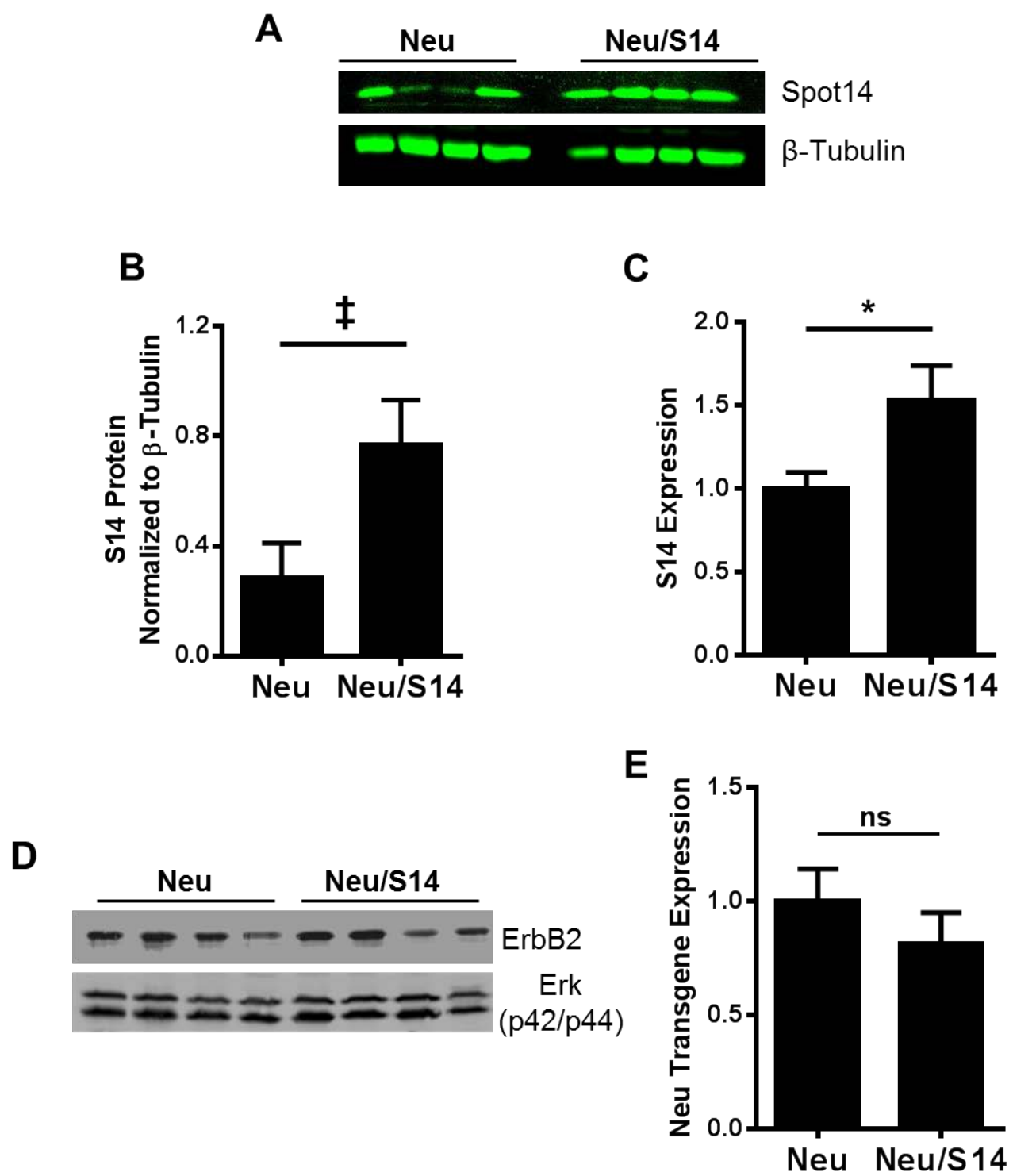

Supplement: Supplementary file 5 — Additional file 5: Gas chromatography-mass spectrometry (GCMS) tumor fatty acids. This file contains a table of the fatty acids analyzed in tumors from polyomavirus middle T antigen (PyMT) (n = 6), PyMT/S14−/− (n = 6), Neu (n = 8), and Neu/S14 (n = 7) mice. Included are the fatty acid chain lengths and saturation, the measured fatty acids in ng/mg tissue, the ratios of each group to its respective control, and the P-values of the differences between groups. (PDF 54 KB) [file 13058_2014_481_MOESM5_ESM.pdf]

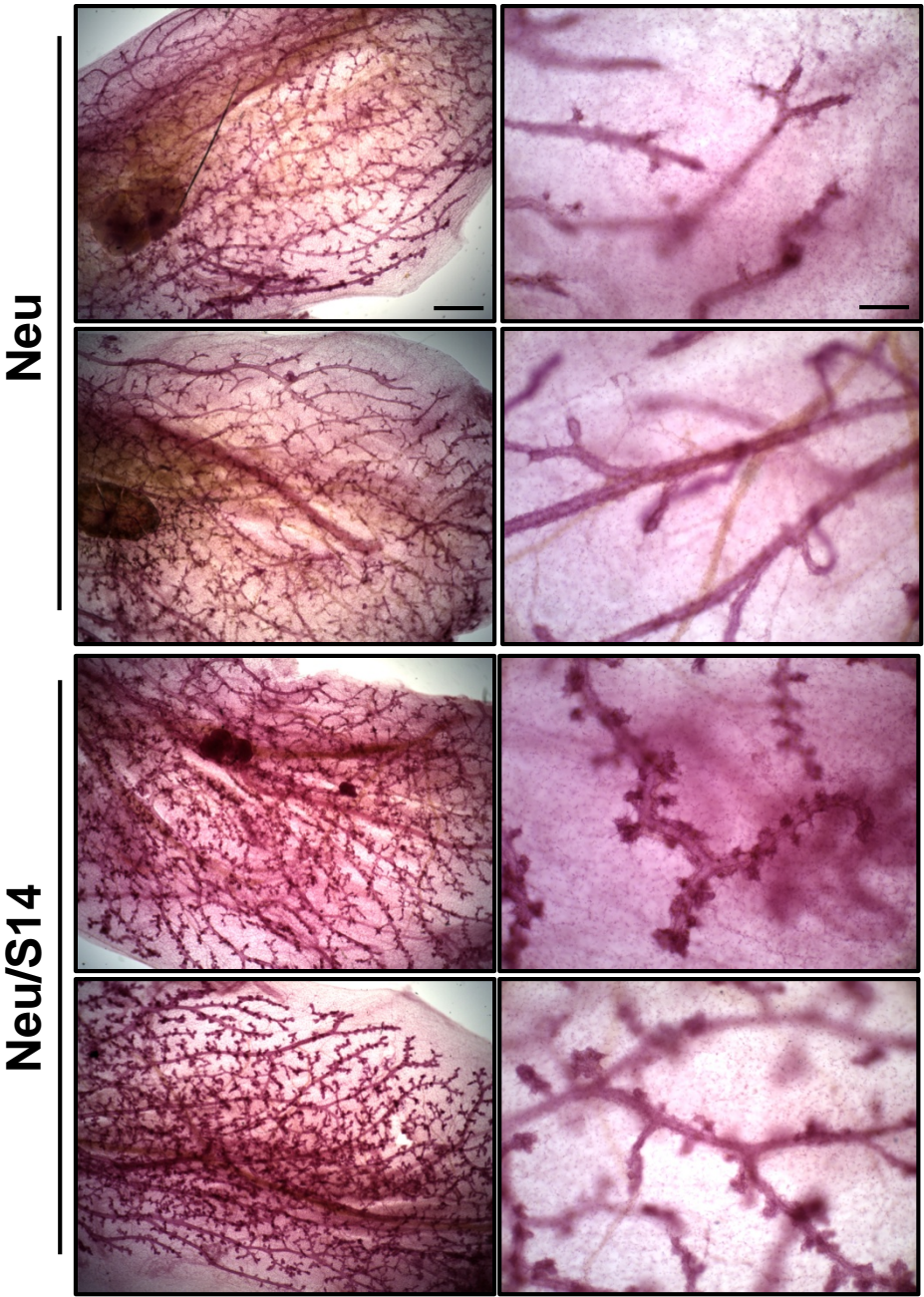

Supplement: Supplementary file 10 — Additional file 10: MG whole mounts Neu and Neu_S14. This file contains images of whole mounted mammary glands from two each of Neu and Neu/S14 mice at 10 months of age and in diestrus. Scale for left column (2x) is 1 mm, scale for right column (10x) is 200 μm. (PDF 642 KB) [file 13058_2014_481_MOESM10_ESM.pdf]

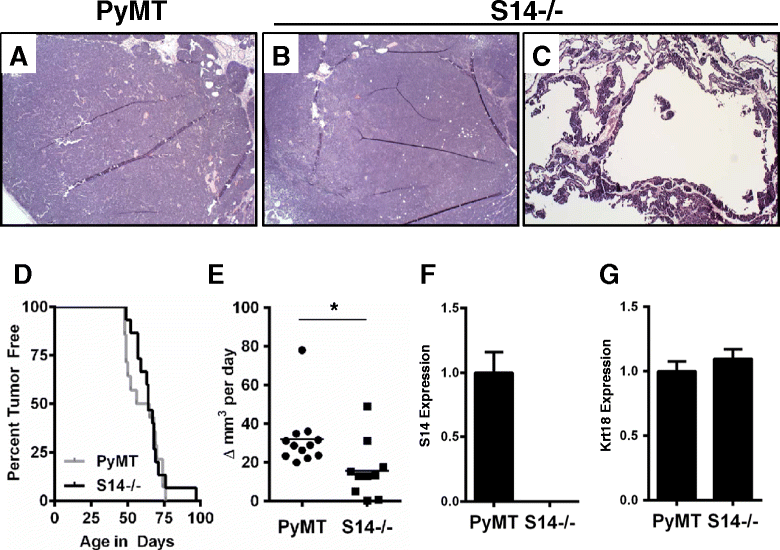

Supplement: Supplementary file 11 — Authors’ original file for figure 1 [file 13058_2014_481_MOESM11_ESM.gif]

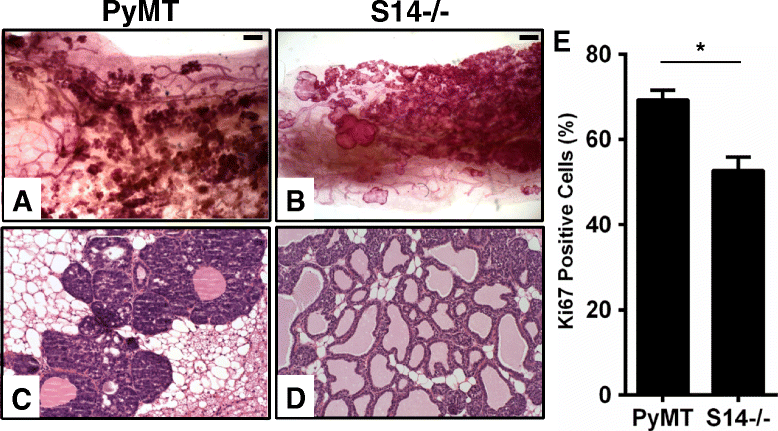

Supplement: Supplementary file 12 — Authors’ original file for figure 2 [file 13058_2014_481_MOESM12_ESM.gif]

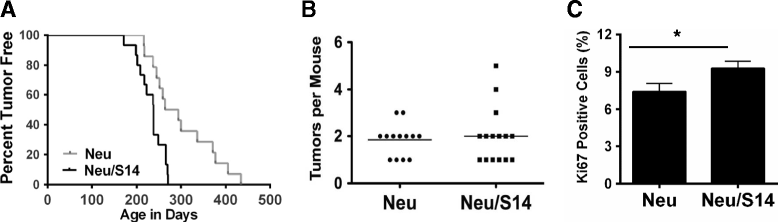

Supplement: Supplementary file 13 — Authors’ original file for figure 3 [file 13058_2014_481_MOESM13_ESM.gif]

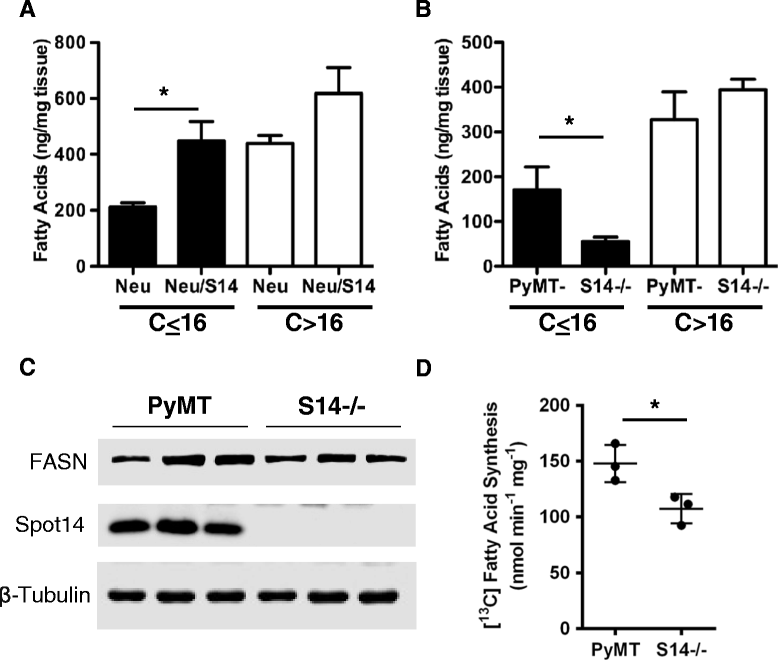

Supplement: Supplementary file 14 — Authors’ original file for figure 4 [file 13058_2014_481_MOESM14_ESM.gif]

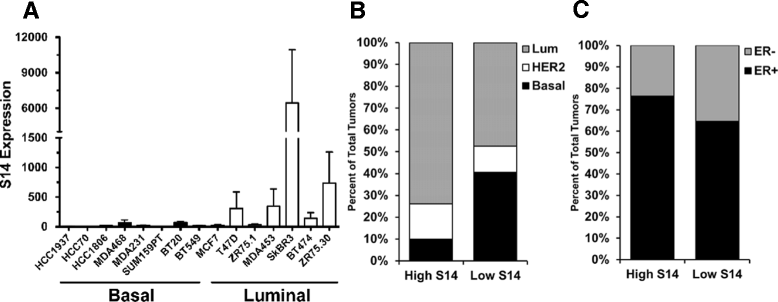

Supplement: Supplementary file 15 — Authors’ original file for figure 5 [file 13058_2014_481_MOESM15_ESM.gif]

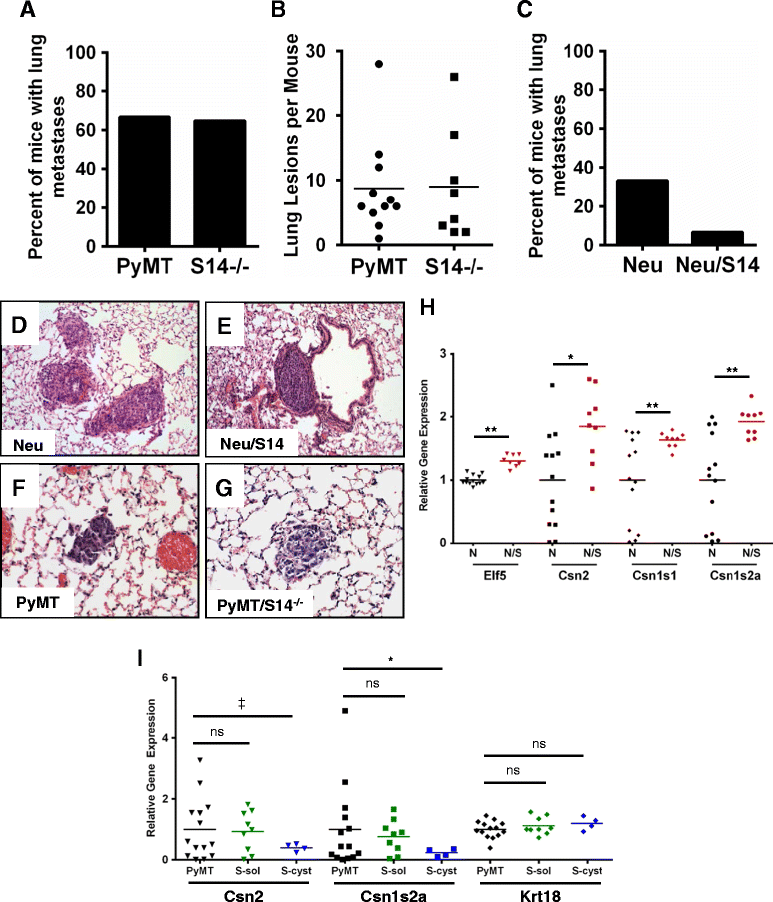

Supplement: Supplementary file 16 — Authors’ original file for figure 6 [file 13058_2014_481_MOESM16_ESM.gif]

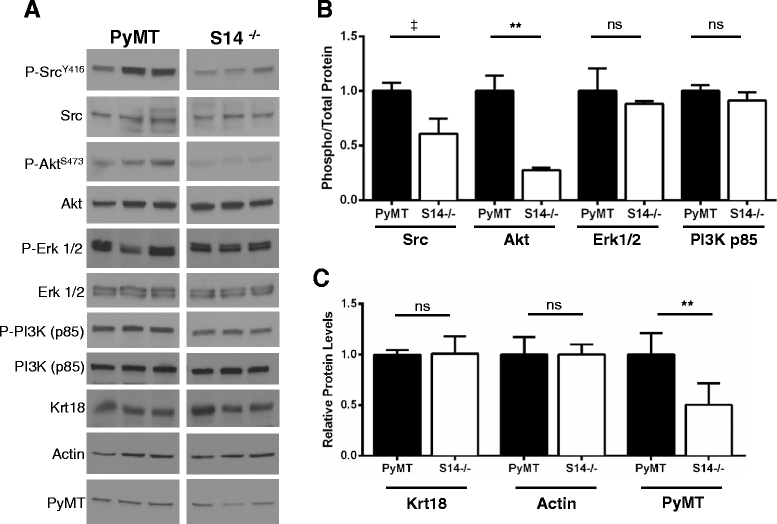

Supplement: Supplementary file 17 — Authors’ original file for figure 7 [file 13058_2014_481_MOESM17_ESM.gif]
