# Supplementary material for: Modulation of tumor fatty acids, through overexpression or loss of thyroid hormone responsive protein spot 14 is associated with altered growth and metastasis
Source: Breast Cancer Res. 2014 Dec 4;16:481. doi: 10.1186/s13058-014-0481-z (PMC4303195; doi:10.1186/s13058-014-0481-z)
Supplement: Supplementary file 2 — Additional file 2: Public microarray datasets. This file contains the GEO ID (if applicable), the number of tumors with low or high S14 expression, the array platform used, the PubMed ID references for each dataset, and the specific analysis for which each dataset was used. (PDF 29 KB) [file 13058_2014_481_MOESM2_ESM.pdf]

| Gene Name and Symbol | Primer Sequence or Catalog Number |
| --- | --- |
| human ThyroidHormone Responsive Protein Spot14 (S14, THRSP) | Thermo Scientific AX-019569-00-0100 |
| human Actin | Applied Biosystems Hs00357333_g1 |
| mouse ThyroidHormone Responsive Protein Spot14 (S14, THRSP) | F-5’-CAG CGA GGC TGA GAA GCA C-3’<br>R-5’-TCC AGC TCC TCCGAG ATC CT-3’<br>Probe-FAM-CTGCTG AAA CGG AGG AGGCCT TGG |
| S14HA (transgene) | F-5’-CAG CGA GGC TGA GAA GCA C-3’<br>R-5’-TAGTVTGGGACGTCGTATGGGTA-3’<br>Probe-FAM-CTGCTG AAA CGG AGG AGGCCT TGG |
| β-Casein(Csn2) | F-5’-GCT CCA GGC TAA AGT TCA CTC C-3’<br>R-5’-GGT TTG AGC CTG AGC ATA TGG-3’<br>Probe-FAM-CAT CCA GTC ACA GCC CCA GGC |
| Keratin 18 (Krt18) | Applied Biosystems Mm01601704_g1 |
| γ-Casein (Csn1s2a) | Applied Biosystems Mm00839343_m1 |
| Arginase 2 (Arg2) | Applied Biosystems Mm00477592_m1 |
| Butyrophilin 1a1 (Btn1a1) | Applied Biosystems Mm00516333_m1 |
| Cholecystokinin (Cck) | Applied Biosystems Mm00446170_m1 |
| Cytoplasmic polyadenylation element binding protein 2 (Cpeb2) | Applied Biosystems Mm00616243_m1 |
| L-amino acid oxidase 1 (Lao1) | Applied Biosystems Mm00192263_m1 |
| Lymphoid enhancer binding factor 1 (Lef1) | Applied Biosystems Mm00550265_m1 |
| TGFbeta receptor III (Tgfbr3) | Applied Biosystems Mm00803538_m1 |
| Aldolase A (Aldoa) | Applied Biosystems Mm00833172_g1 |
| Phosphofructokinase, liver B-type (Pfkl) | Applied Biosystems Mm00435587_m1 |
| Hexokinase II (Hk2) | Applied Biosystems Mm00443385_m1 |
| E74-like factor 5 (Elf5) | Applied Biosystems Mm01340245_m1 |
| Homer homolog 2 (Homer2) | F-5'-ACA GCA CTA TCA CCC CGA AC-3'<br>R-5'-GAC GCT GGA TGC TTG GGA AT-3' |
| Glycerophosphodiester phosphodiesterase domain containing 3 (Gdpd3) | F-5'-CTC TCC TGT ACT TTG TTC TGC C-3'<br>R-5'-CCA GGC GGA TAG GGA AGA C-3' |
| lysophosphatidic acid acyltransferase, alpha (Agpat1) | F-5'-TAA GAT GGC CTT CTA CAA CGG C-3'<br>R-5'-CCA TAC AGG TAT TTG ACG TGG AG-3' |
