# Supplementary material for: Modulation of tumor fatty acids, through overexpression or loss of thyroid hormone responsive protein spot 14 is associated with altered growth and metastasis
Source: Breast Cancer Res. 2014 Dec 4;16:481. doi: 10.1186/s13058-014-0481-z (PMC4303195; doi:10.1186/s13058-014-0481-z)
Supplement: Supplementary file 3 — Additional file 3: QPCR validation of microarray data. This file contains a table showing qPCR results from microarray targets. The mean expression values with standard error of the mean (SEM) for Neu (n = 13) and Neu/S14 (n = 14) and polyomavirus middle T antigen (PyMT) (n = 14) and S14−/− (n = 9) tumors are shown, with fold changes (Neu/S14 versus Neu or S14−/− vs PyMT) and P-values. (PDF 34 KB) [file 13058_2014_481_MOESM3_ESM.pdf]

Additional File 2

GEO ID	Total Tumors	Low S14	High S14	Platform	PMID	Analysis Performed
GSE19615	115	**30**	**30**	HG-U133P2	20098429	ER Correlation
GSE6532	414	**102**	**102** (97 w/ ER data)	HG-U133A, HG-U133B, HG-U133P2	20479250, 18498629, 17401012	ER Correlation
GSE20685	327	**82**	**82**	HG-U133P2	21501481	ER Correlation; Intrinsic Subtype Correlation
GSE1456	159	**40** (32 w/subtype data)	**40** (23 w/ subtype data)	HG-U133A, HG-U133B	16280042	Intrinsic Subtype Correlation
N/A (avail. from Oncomine)	295	**74**	**74**	Printed Microarray	12490681, 11283592	ER Correlation
GSE4922	249	**63**	**63**	HG-U133A, HG-U133B	17079448	ER Correlation
GSE21653	266	**67** (55 w/ ER data; 58 w/ subtype data)	**67** (58 w/ ER data; 59 w/ subtype data)	HG-U133P2	20490655, 22110708	ER Correlation; Intrinsic Subtype Correlation
GSE22226	130	**33** (32 w/ ER data; 32 w/ subtype data)	**33** (32 w/ ER data; 28 w/ subtype data)	Agilent Human Genome 44K	22198468	ER Correlation; Intrinsic Subtype Correlation
