# Supplementary material for: Modulation of tumor fatty acids, through overexpression or loss of thyroid hormone responsive protein spot 14 is associated with altered growth and metastasis
Source: Breast Cancer Res. 2014 Dec 4;16:481. doi: 10.1186/s13058-014-0481-z (PMC4303195; doi:10.1186/s13058-014-0481-z)
Supplement: Supplementary file 4 — Additional file 4: Transgene levels in Neu and Neu_S14 Tumors. (A) Immunoblot of S14 in tumors from Neu and Neu/S14 mice, with β-Tubulin as a loading control. (B) Quantification of S14 protein levels normalized to B-Tubulin; P = 0.06. (C) QPCR analysis of S14 gene expression in tumors from Neu (n = 13) and Neu/S14 (n = 12) mice. P = 0.025. (D) Immunoblot analysis of Neu (ErbB2) in tumors from Neu and Neu/S14 mice, with Erk as a loading control. (E) QPCR analysis of Neu transgene expression in tumors from Neu (n = 13) and Neu/S14 (n = 12) mice. (PDF 41 KB) [file 13058_2014_481_MOESM4_ESM.pdf]

	Mean		SEM		Fold (Neu/S14:Neu)	p-value
	Neu	Neu/S14	Neu	Neu/S14		
Arg2	306.8	814.7	41.6	147.4	2.66	0.002
Btn1a1	14113.8	27180.5	3777.0	3873.8	1.93	0.016
Cck	863.8	3488.7	287.6	816.3	4.04	0.005
Cpeb2	2888.7	1578.7	254.9	83.5	0.55	2.72E-05
Lao1	2464.5	7081.8	689.1	1874.7	2.87	0.020
Lef1	444.1	816.8	90.1	111.5	1.84	0.010
Tgfbr3	1367.3	945.5	140.9	59.0	0.69	0.014
Elf5	17693.5	25001.8	1206.4	2271.0	1.41	0.005

	Mean		SEM		Fold ($S14^{-/-}$:PyMT)	p-value
	PyMT	$S14^{-/-}$	PyMT	$S14^{-/-}$		
Aldoa	153654.0	115236.1	11400.0	6818.2	0.75	0.017
Pfkl	26081.0	18844.0	2579.3	1825.7	0.72	0.036
Hk2	2436.1	1338.0	365.4	238.9	0.55	0.017
Homer2	28320.1	57124.8	4176.2	7563.6	2.02	0.009
Gdpd3	17484.2	7672.9	2844.2	1010.3	0.44	0.021
Agpat1	23877.8	15937.2	2126.3	1048.6	0.67	0.006
