# Supplementary material for: Modulation of tumor fatty acids, through overexpression or loss of thyroid hormone responsive protein spot 14 is associated with altered growth and metastasis
Source: Breast Cancer Res. 2014 Dec 4;16:481. doi: 10.1186/s13058-014-0481-z (PMC4303195; doi:10.1186/s13058-014-0481-z)
Supplement: Supplementary file 6 — Additional file 6: Effects of S14 on fatty acid synthesis enzymes. Gene expression and protein levels of ATP-citrate lyase (ACLY), acetyl Co-A carboxylase (ACC), and fatty acid synthase (FASN) in Neu (n = 14) and Neu/S14 (n = 15) tumors (A and B, respectively) and polyomavirus middle T antigen (PyMT) (n = 4) and S14−/− (n = 4) tumors (C and D, respectively). Immunoblots were quantified for Neu and Neu/S14 as well as PyMT and S14−/− tumors in E and F, respectively. ns, not significant. (PDF 34 KB) [file 13058_2014_481_MOESM6_ESM.pdf]

Additional File 3

Fatty Acid Chain Length and Saturation	Fatty Acids (ng per mg tissue)		Ratio S14-/- to PyMT	p-value	Fatty Acids (ng per mg tissue)		Ratio Neu/S14 to Neu	p-value
	PyMT	S14-/-			Neu	Neu/S14		
**10:0**	0.37	nd	0.00	0.113	5.65	19.14	3.39	0.068
**12:0**	2.75	nd	0.00	0.028	6.12	9.12	1.49	0.349
**14:0**	16.90	4.95	0.29	0.005	23.52	45.78	1.95	0.026
**14:1**	0.41	0.28	0.69	0.209	1.06	3.89	3.68	0.015
**16:0**	120.87	34.44	0.28	0.027	157.33	328.56	2.09	0.010
**16:1**	29.10	15.66	0.54	0.11	17.73	93.91	5.30	0.103
**18:0**	15.51	12.02	0.77	0.234	143.39	202.74	1.41	0.062
**18:1**	328.43	197.66	0.60	0.191	154.88	292.21	1.89	0.026
**18:2**	236.79	116.73	0.49	0.178	100.49	227.83	2.27	0.027
**18:3**	12.28	6.10	0.50	0.183	0.71	0.67	0.95	0.817
**20:4**	47.10	61.99	1.32	0.026	39.54	42.94	1.09	0.868
